# Acetyl-L-Carnitine Induces Autophagy to Promote Mouse Spermatogonia Cell Recovery after Heat Stress Damage

**DOI:** 10.1155/2021/8871328

**Published:** 2021-01-18

**Authors:** Na Qiao, Hanming Chen, Peiquan Du, Zhenlong Kang, Congying Pang, Bingxian Liu, Qiwen Zeng, Jiaqiang Pan, Hui Zhang, Khalid Mehmood, Zhaoxin Tang, Ying Li

**Affiliations:** ^1^College of Veterinary Medicine, South China Agricultural University, Guangzhou 510642, China; ^2^Faculty of Veterinary & Animal Sciences, The Islamia University of Bahawalpur, Bahawalpur 63100, Pakistan

## Abstract

Acetyl-L-carnitine (ALC) is an effective substrate for mitochondrial energy metabolism and is known to prevent neurodegeneration and attenuate heavy metal-induced injury. In this study, we investigated the function of ALC in the recovery of mouse spermatogonia cells (GC-1 cells) after heat stress (HS). The cells were randomly divided into three groups: control group, HS group (incubated at 42°C for 90 min), and HS + ALC group (treatment of 150 *μ*M ALC after incubated at 42°C for 90 min). After heat stress, all of the cells were recovered at 37°C for 6 h. In this study, the content of intracellular lactate dehydrogenase (LDH) in the cell supernatant and the malondialdehyde (MDA) levels, catalase (CAT) levels, and total antioxidant capacity (T-AOC) were significantly increased in the HS group compared to the CON group. In addition, the mitochondrial membrane potential (MMP) was markedly decreased, while the apoptosis rate and the expression of apoptosis-related genes (Bcl-2, Bax, and caspase3) were significantly increased in the HS group compared to the CON group. Furthermore, the number of autophagosomes and the expression of autophagy-related genes (Atg5, Beclin1, and LC3II) and protein levels of p62 were increased, but the expression of LAMP1 was decreased in the HS group compared to the CON group. However, treatment with ALC remarkably improved cell survival and decreased cell oxidative stress. It was unexpected that levels of autophagy were markedly increased in the HS + ALC group compared to the HS group. Taken together, our present study evidenced that ALC could alleviate oxidative stress and improve the level of autophagy to accelerate the recovery of GC-1 cells after heat stress.

## 1. Introduction

Global warming is causing more and more organisms to be under heat stress which influences animal growth, development, and reproduction [1, 2]. The male reproduction is one of the most sensitive processes to be damaged by heat stress because the optimal temperature for spermatogenesis is slightly lower than that of the body [3, 4]. Among the germ cells, the pachytene spermatocytes and the sperm are very vulnerable to heat [5–7]. Some studies have correlated that heat stress can affect spermatogenesis to fail by increasing the apoptosis of testicular germ cells, and it can also lead to the fragmentation of sperm DNA [8, 9]. In marked contrast, spermatogonia appear relatively resilient to heat stress [10]. Heat-resistant spermatogonia can gradually recover and differentiate into new sperm after heat stress, which can gradually improve sperm quality and reduce the germ cell apoptosis [7]. So the resilience of spermatogonia plays a vital role in the recovery of sperm quality after heat stress. However, few studies have focused on the recovery capability of spermatogonia after heat stress.

Autophagy is a cytoprotective mechanism for cells to gain energy by phagocytosing organelles and proteins under various stress [11–13]. The function of autophagy in cell survival is complex. On the one hand, autophagy can degrade cellular components to maintain cell survival under stress. On the other hand, excessive autophagy has also been shown to promote cell apoptosis under certain conditions. A previous study has reported that heat stress increased autophagy in the testes of mice [14]. Furthermore, oxidative stress is considered to be one of the leading causes of damages of germ cells in heat stress [9]. Nonetheless, when spermatogonia are exposed to heat, the underlying mechanisms of autophagy remain unclear.

Acetyl-L-carnitine (ALC) is a natural substance found in the body. It is an effective substrate for mitochondrial energy metabolism and plays a crucial role in the oxidation of fatty acid [15, 16]. ALC can prevent neurodegeneration and attenuate heavy metal-induced injury through improve mitochondrial functions and antioxidant capacity [17–19]. In addition, many clinical studies have shown that oral ALC increases the motility of spermatozoa, the velocity of spermatozoa, and the ratio of living spermatozoa [20, 21]. However, the protective effect of ALC on GC-1 cells after heat treatment remains unclear. In this study, we explored the ability of ALC to promote mouse spermatogonia cell recovery after heat stress damage and demonstrated its ability to antioxidant functions and upregulation of autophagy.

## 2. Materials and Methods

### 2.1. Cell Line and Reagents

The mouse-derived spermatogonia cell line (GC-1 cell line) was bought from American Type Culture Collection (ATCC) and maintained in DMEM medium (Gibco, NY, USA) supplemented with 10% FBS (Gibco, NY, USA). Primary antibodies, anti-Beclin1 and anti-LC3, were from Sigma-Aldrich; anti-GAPDH was from Bioss Biotechnology. Secondary antibodies for immunoblot analysis were from Bioss Biotechnology. ALC was from Santa Cruz Biotechnology. Cell counting kit-8 (CCK-8) solution was from Dojindo Molecular Technologies Institute. Catalase (CAT) (A007-1-1), malondialdehyde (MDA) (A003-1-2), and total antioxidant capacity (T-AOC) (A015-1-2) assay kits were from Nanjing Jiancheng Bioengineering Institute (http://www.njjcbio.com/product.asp?cid=27&sort=saleorder). 5,5′,6,6′-Tetrachloro-1,1′,3,3′-tetraethylbenzimidazolyl-carbocyanine iodide (JC-1), monodansylcadaverine (MDC), 4′,6-diamidino-2-phenylindole (DAPI), and acridine orange/ethidium bromide (AO/EB) staining Kit were from Beyotime Biotechnology (https://www.beyotime.com/index.htm). Trizol reagent and Prime Script reverse transcriptase reagent kit were from TaKaRa Biotechnology.

### 2.2. Cell Culture and Heat-Shock Treatment

Cells were seeded and divided into three treatment groups: CON group, cells without any treatment; HS group, cells treatment with heat stress for 90 min; and HS-ALC group, cells treated with 150 *μ*M ALC after heat stress. The CON group was placed into an incubator at 37°C, whereas the experimental groups were incubated at 42°C incubator for heat stress for 90 min. ALC was dissolved in DMEM to yield a 150 mM stock solution and was dissolved in culture medium to yield the working solution with 150 *μM* ALC, while the same volume of DMEM was added to the control and the HS groups. After heat stress, all of the cells were recovered in a 37°C incubator for 6 h.

### 2.3. Cell Viability Assay

GC-1 cells were seeded in 96-well plates at a density of 5000 cells per well. Then, 10 *μ*L CCK-8 solution was added to each well after cells were treated according to the experiment. After cells were maintained at 37°C for 1 h, OD values were measured at 450 nm using microplate spectrophotometer.

### 2.4. Lactate Dehydrogenase (LDH) Assay

The LDH content in cell growth medium at 6 h after heat stress treatment was assayed using an automatic biochemical analyzer (BS-380; Mindray, Shenzhen, China).

### 2.5. Determination of Oxidative Stress Indicators

Firstly, 0.25% trypsin was used to digest the cells, collecting 10^7^ cells. Then, 1 mL of normal saline was assed to the cells, and the cells were broken with homogenizer. After further centrifugation at 1000 × g for 15 min, the supernatant is collected to determine CAT activity, MDA levels, and T-AOC activity. All experimental procedures were performed according to the reagent instructions.

### 2.6. Mitochondria Membrane Potential (MMP) Assay

MMP was evaluated by using the JC-1 solution according to the manufacturer's instruction. In brief, cells were collected and incubated with JC-1 staining solution inside the CO_2_ incubator for 30 min, then rinsed and resuspended in JC-1 buffer. Finally, quantification by flow cytometry (CytoFLEX; Beckmen) detected mitochondria containing red JC-1 aggregates in PE (excitation: 490 nm; emission: 530 nm) channel and green JC-1 monomers in the FITC channel (excitation: 525 nm; emission: 590 nm).

### 2.7. Quantitative PCR Analysis

Quantitative PCR analysis was performed according to a method described previously [22]. In briefly, the cells were harvested in Trizol reagent for total RNA extraction. Prime Script reverse transcriptase reagent kit was used to reverse transcribe total RNAs into complementary DNA. PCRs were set up using a Detection System (Light Cycler 480II; Roche, USA). Relative mRNA levels of target genes were analyzed by the 2^-△△Ct^ method. GAPDH was used as the reference gene. Specific primer sequences are listed in Table 1.

### 2.8. Monodansylcadaverine (MDC) Staining

GC-1 cells were harvested and incubated with MDC at 37°C for 30 min. Then, cells were washed with PBS. Finally, the image of the cells was conducted under a fluorescence microscope (DM i8; Lecia, Germany) and observed at 512 nm when excited at 355 nm.

### 2.9. Western Blot Analysis

Total proteins were homogenized in RIPA lysis buffer containing 1 mM phenylmethanesulfonyl fluoride (PMSF). Then, proteins were quantified by the BCA assay kit. Equal proteins (10 *μ*g) of each sample were loaded onto SDS-PAGE gels. The blot was transferred onto polyvinylidene difluoride (PVDF) membranes and blocked with 5% nonfat milk for 1 h at room temperature and incubated with primary antibodies for 16 h at 4°C. Following the incubation of the secondary antibody, the blot was developed by the electrochemiluminescence liquid (ECL) method by using the Image Lab software (Biorad, CA, USA). The density of the blot was analyzed by Image J.

### 2.10. Immunofluorescence Assay

GC-1 cells were fixed in 4% paraformaldehyde and blocked with 5% BSA. Cells then were incubated with rabbit anti-LC3II antibody (1 : 200) for 16 h at 4°C and washed with PBS. Secondary antibodies conjugated to FITC were used. Cell nuclei were stained using DAPI. The image was taken by a Leica SP8 confocal microscope (Leica, Germany). Finally, the photographs were analyzed by ImageJ.

### 2.11. Apoptosis Analysis

After cells were treated according to the experiment, cells were harvested and resuspended in PBS. Then, cells were stained using the AO/EB staining Kit according to the instructions. The cells were scanned at emission/excitation wavelengths of 488/512 nm and 510/595 nm, respectively. Finally, cells were analyzed with a fluorescent microscope (DM i8; Lecia, Germany).

### 2.12. Statistical Analysis

Data were analyzed using SPSS 18.0. Student's *t*-test or one-way analysis of variance (ANOVA) was used to analyzed the *P* value (∗*P* < 0.05; ∗∗*P* < 0.01). Data in this study were expressed as the mean ± standard deviation (SD). Graphs are plotted using software GraphPad Prism 6.

## 3. Results

### 3.1. Effect of ALC on Cell Proliferation and LDH Release in GC-1 Cells after Heat Stress

**Figure 1:**
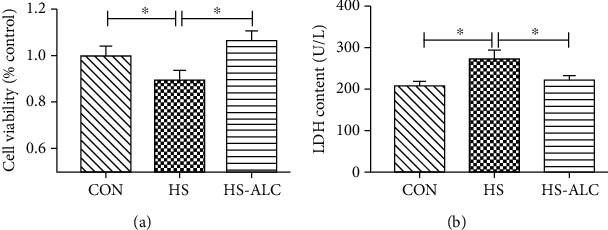
Effect of heat stress and ALC on cell viability and LDH release: (a) cell viability of GC-1 cells detected by CCK-8; (b) LDH content in supernatants. The data are presented as the mean ± SD, *n* = 3, ^∗^*P* < 0.05, ^∗∗^*P* < 0.01. CON: control group; HS: recover at 6 h; HS-ALC: recover at 6 h with ALC.

Results of CCK-8 showed that cell viability was markedly reduced in the HS group (*P* < 0.05) compared to the CON group. However, the decrease of cell viability induced by heat stress was obviously reversed by ALC (*P* < 0.05) (Figure 1(a)). Besides the cell viability, GC-1 cell injuries were also measured by determining LDH activities in the media after 12 h culture. LDH leakage from GC-1 cells was significantly elevated in the HS group compared to the CON group (*P* < 0.05) (Figure 1(b)), but this increase was remarkedly reduced after the combined administration of ALC (*P* < 0.05) (Figure 1(b)). In addition, no obvious differences in LDH activities were observed between the CON group and the HS-ALC group (*P* > 0.05) (Figure 1(b)).

### 3.2. Oxidative Damage and Antioxidant Ability in GC-1

**Figure 2:**
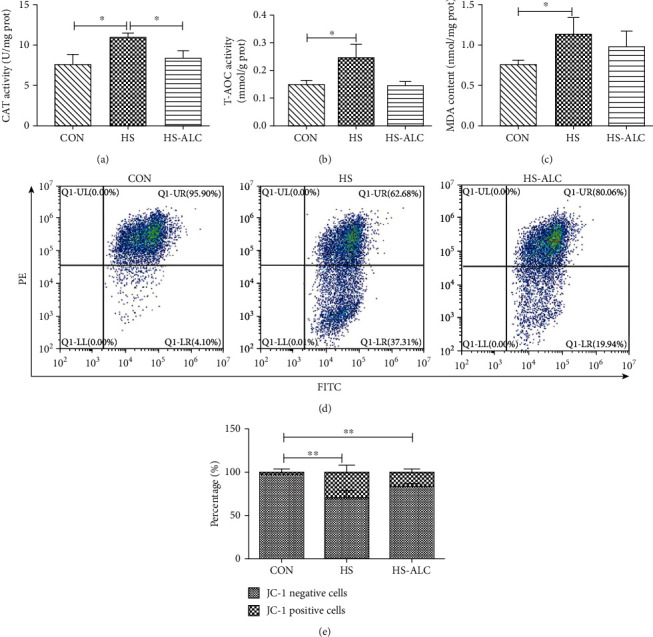
Cell damage associated with heat stress in the recovery phase: (a) CAT activity; (b) T-AOC activity; (c) MDA level; (d, e) MMP, the flow cytometry assay was carried out for detecting MMP. The data are presented as the mean ± SD, *n* = 3, ^∗^*P* < 0.05, ^∗∗^*P* < 0.01. CON: control group; HS: recover at 6 h; HS-ALC: recover at 6 h with ALC.

The activities of CAT and T-AOC and the concentration of MDA were measured to investigate the oxidative damage and antioxidant ability in GC-1 cells. In Figures 2(a)–2(c), CAT and T-AOC activities and MDA levels were increased notably in the HS group compared to the CON group (*P* < 0.05). However, the elevated CAT activities by heat stress were markedly reversed by ALC (*P* < 0.05) (Figure 2(a)). In addition, T-AOC activities and MDA levels slightly decreased in the HS-ALC group were slightly lower than that in the HS group, but there were no significant differences between the two groups (*P* > 0.05) (Figures 2(b) and 2(c)). Besides, exposure to heat stress significantly decreased the MMP compared to the CON group (*P* < 0.01) (Figures 2(d) and 2(e)). Treatment with the ALC slightly blocked the heat stress-induced decline of the MMP in GC-1 cells (*P* > 0.05) (Figures 2(d) and 2(e)).

### 3.3. Effect of ALC on Autophagy in GC-1 Cells

**Figure 3:**
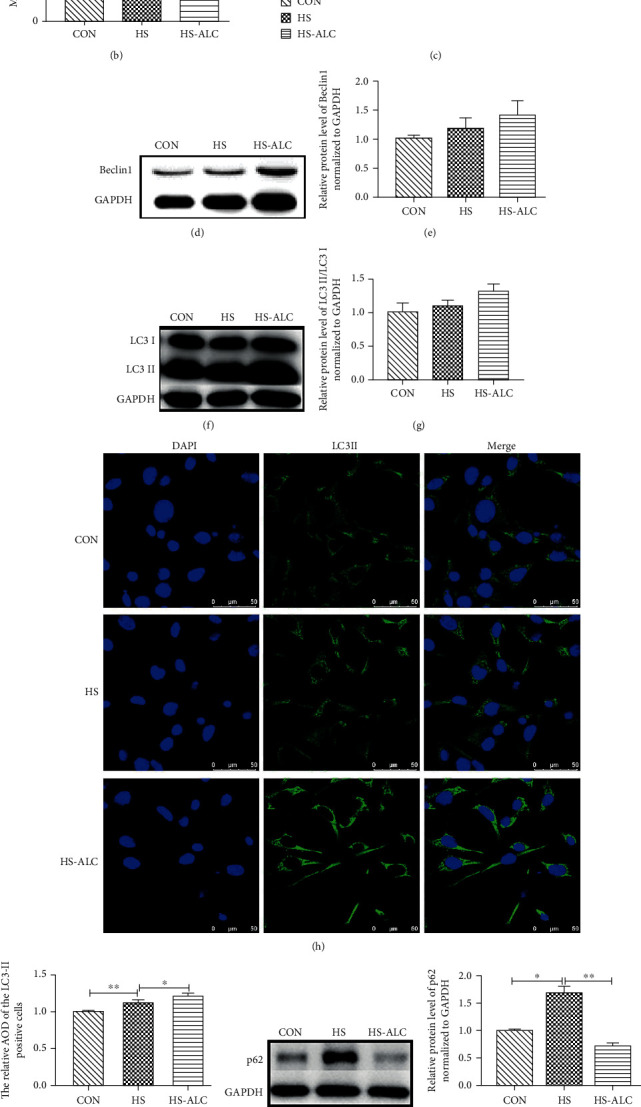
Effect of ALC on heat stress-induced autophagy in GC-1 cells. (a) Detection of autophagosomes using monodansylcadaverine (MDC) staining. (b) Percentages of MDC-positive cells. (c) mRNA levels of Atg5, Beclin1, and LC3II in GC-1 cells. (d, e) Western blot analysis of LC3II/LC3I protein expression in GC-1 cells. (f, g) Western blot analysis of Beclin1 protein expression in GC-1 cells. (h) The immunofluorescence of LC3II in GC-1 cells. (i) The relative AOD of the LC3II positive cells. (j, k) Western blot analysis of p62 protein expression in GC-1 cells. (l) The mRNA levels of lysosomal biogenesis genes were determined using RT-PCR. The data are presented as the mean ± SD, *n* = 3, ^∗^*P* < 0.05, ^∗∗^*P* < 0.01. CON: control group; HS: recover at 6 h; HS-ALC: recover at 6 h with ALCAR.

MDC is a maker of acidic vesicles which is used to monitor later-stage autophagy. MDC staining was applied to observe GC-1 cells autophagy changes on the condition of heat. As shown in Figures 3(a) and 3(b), the number of MDC positive staining cells in the HS group and the HS-ALC group was significantly increased compared to the CON group (*P* < 0.05). Meanwhile, there was a markedly increased number of MDC positive staining cells in the HS-ALC group compared to the HS group (*P* < 0.05) (Figures 3(a) and 3(b)). What is more, heat exposure significantly increased mRNA levels of Atg5, Beclin1, and LC3II in GC-1 cells (*P* < 0.01) (Figure 3(c)). However, ALC treatment further increased the expression of Atg5, Beclin1, and LC3II mRNA in heat treatment cells (*P* < 0.05) (Figure 3(c)). Six hours after heat stress, we found that the protein levels of Beclin1 and LC3II/LC3I were remarkedly higher compared to the CON group (*P* < 0.01) (Figures 3(e)–3(g)). Immunofluorescence data illustrated that the levels of LC3II were notably increased in the HS-ALC group compared to the CON group (*P* < 0.01), whereas the levels of LC3II were markedly increased in the HS-ALC group compared to the HS group (*P* < 0.05) (Figures 3(h) and 3(i)). Furthermore, the protein levels of p62 markedly upregulated in the HS group compared to the CON group and the HS-ALC group (*P* < 0.05) (Figures 3(j) and 3(k)). The mRNA levels of LAMP1 were markedly decreased in the HS group compared to the Con group (*P* < 0.01) (Figure 3(l)). Additionally, the expressions of Mcoln1, LAMP1, and LAMP2 were significantly increased in the HS-ALC group compared to the HS group (*P* < 0.01) (Figure 3(l)).

### 3.4. Effect of ALC on Apoptosis in GC-1

**Figure 4:**
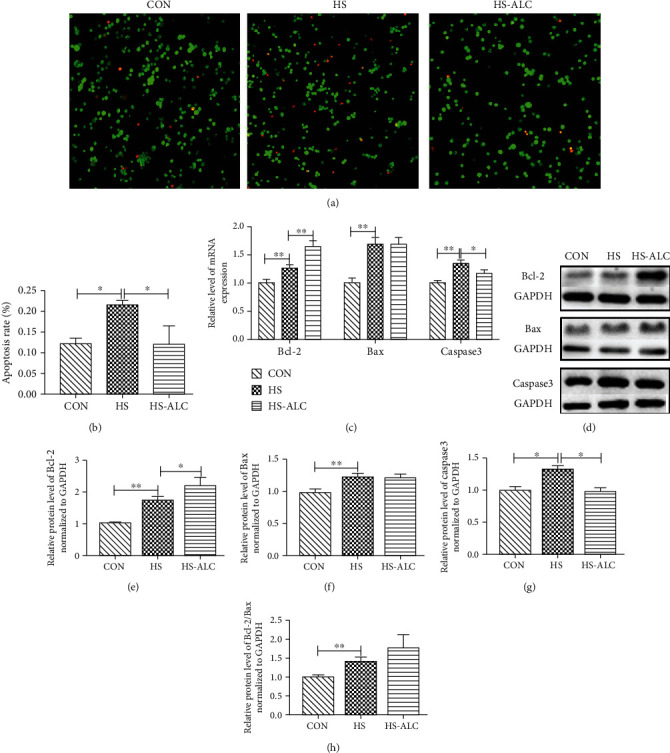
Effect of ALC on heat stress-induced apoptosis in GC-1 cells. (a) The cells were labeled with AO/EB and then analyzed by fluorescence microscope. (b) Data were expressed as the percent of apoptotic cells obtained from the histogram statistics all quantitative. (c) The mRNA levels of Bcl-2, Bax, and caspase3. (d) Immunoblot analysis of apoptosis-related proteins. (e, f) The protein levels of Bcl-2, Bax, caspase3, and Bcl-2/Bax. The data are presented as the mean ± SD, *n* = 3, ^∗^*P* < 0.05, ^∗∗^*P* < 0.01. CON: control group; HS: recover at 6 h; HS-ALC: recover at 6 h with ALCAR.

As shown in Figures 4(a) and 4(b), the rate of apoptotic cells was remarkably decreased in the CON group and the HS-ALC group compared to the HS group (*P* < 0.05). Additionally, the mRNA and protein levels of Bcl-2, Bax, caspase3 were significantly increased in the HS group compared to the CON group (*P* < 0.05) (Figures 4(c)–4(g)). The addition of ALC significantly upregulated the mRNA and protein levels of Bcl-2 and downregulated the mRNA and protein levels of caspase3 (*P* < 0.05) (Figures 4(c)–4(g)). Furthermore, the protein levels of Bcl-2/Bax were significantly increased in the HS group compared to the CON group (*P* < 0.05) (Figures 4(d) and 4(h)).

## 4. Discussion

With global warming, all living beings are facing the adverse effects of it, and especially, the male animals are facing enormous challenges in reproduction. Usually, the harmful effect of heat stress on spermiogenesis in the mammal is mainly through inducing oxidative damage, disturbing the mitochondrial function, and increasing cell mortality [23]. Several studies have demonstrated the antioxidative and mitochondrial membrane stabilizing properties of ALC [17, 24]. However, little evidence is accessible about the role of ALC in promoting spermatogonia recovery from the damage of heat stress. In this study, we found that ALC attenuated HS-induced viability and apoptosis by reducing oxidative stress and upregulating autophagy after heat stress.

To investigate the protective role of ALC, cell viability and LDH leakage were conducted. Previous research has shown that the increased LDH content in cell supernatant is due to the increased cell membrane permeability under heat stress [25]. In this study, we also found the same phenomenon, the ascent of the concentration of LDH in the supernatant after heat stress. However, the LDH concentration decreased significantly when ALC was added to the cell medium before heat stress. At the same time, ALC treatment also increases cell viability. These findings show that ALC could protect the integrity of the GC-1 cell membrane to accelerate the recovery of GC-1 cells after thermal stress. Furthermore, we investigated the effect of the heat stress and ALC treatment on antioxidation against of GC-1 cells. The activities of intracellular oxidation and antioxidant enzyme reflect the antioxidant capacity of the cells. CAT is considered an essential enzyme in the process of ROS elimination by decomposing hydrogen peroxide to H_2_O and O_2_ [26]. MDA is the final product of lipid peroxidation in cells and is considered a sign of oxidative stress [27]. T-AOC indicates the oxidation resistance capacity of the cell. In the present study, CAT, MDA, and T-AOC levels were markedly increased in GC-1 cells after recovered for 6 h. So we speculated that GC-1 cells promoted cell recovery by increasing the activity of antioxidant enzymes (CAT and T-AOC) after heat stress. Nevertheless, the elevated activity of the antioxidant enzyme was not enough to against effects from heat; MDA was largely accumulated in cell treatment of heat. These finding indicated that GC-1 cells were severely oxidatively damaged, and oxidative stress may play an essential role in heat stress-induced damage. A recent in vivo study showed that ALC treatment significantly suppressed the oxidative damage induced by NaAsO_2_ through maintaining oxidant-antioxidant balance [17]. In this research, we found that ALC has a similar protective function on the GC-1 cells under heat stress. ALC treatment decreased LDH, MDA, and CAT levels in GC-1 cells after heat stress.

When cells are stimulated or damaged, many changes will take place in mitochondrial biological function, such as the change of mitochondrial membrane permeability and the loss of mitochondrial membrane potential [22]. In this study, we found that HS causes a loss of MMP by 35% compared to the control group. Conversely, ALC treatment reduced HS-induced decrease in MMP. The loss of MMP is closely related to programmed cell death and mitochondrial apoptosis [28]. So we examined the expression of apoptosis-related genes. The Bcl-2 protein family plays a decisive role in the regulation of apoptosis, which may either inhibit (Bcl-2) or promote apoptosis (Bax). Cell apoptosis can be evaluated by the ratio of Bcl-2 and Bax [29]. In response to various cellular stresses, the apoptotic factors of the Bcl-2 family change the permeability of the mitochondrial outer membrane and release apoptotic factors into the cytosol [30]. Caspase9 is then activated by these proapoptotic factors [31]. Finally, active caspase9 cleaves and activates the executioner caspase3. In the present study, the apoptotic rate was increased in the HS group compared to the CON group. Moreover, the expression of proapoptotic genes and protein (Bax and caspase3) increased significantly in the HS group compared to the CON group. However, we also found that heat stress increases the expression of apoptosis suppressor gene and protein Bcl-2. In previous studies, Bcl-2 mRNA and protein levels in testes and mouse granulosa cells were also increased after heat stress, but they did not have enough direct evidence to clarify the reason for the rise of Bcl-2 in heat-stressed cells [32–34]. One of these studies suggested that the increasing level of Bcl-2 mRNA expression might be the result of heat shock proteins, which was a self-protection mechanism in granulosa cells [34]. Therefore, we also speculated that the recovery of GC-1 cells after heat stress might be related to the upregulation of antiapoptotic gene Bcl-2. Furthermore, treatment with ALC decreased the apoptotic rate and expression of caspase3 mRNA and protein levels and increased the expression of Bcl-2 mRNA and protein levels in GC-1 cells. Based on these results, it seems possible that ALC might promote the recovery of GC-1 cells after heat stress through increasing Bcl-2 expression and decreasing caspase3 expression.

Autophagy, or type II programmed cell death, is a cellular process to remove the damaged organelles and protein aggregates [35]. During apoptosis, the stimulation of autophagy can be either a protective mechanism or a process that contributes to cell death [36]. Heat stress has become an important issue in stimulating autophagy [37]. Acidic vesicles occur during autophagy, so MDC staining is often used to monitor autophagy. In this study, the results of the MDC staining showed that the number of autophagosomes was markedly increased after cell recovery for 6 h and the number of autophagosomes was further increased while the cells treated with ALC. In addition, there are several genes that are closely related to the process of autophagy. Autophagy-related gene 5 (Atg5) and beclin1 are primary for the process of autophagy. Beclin1 is a platform by binding several cofactors to assemble the class III phosphatidylinositol 3-kinase (PI3K) complex during autophagosome formation [38]. When phagophore is elongating, the Atg5-Atg12 conjugating system combines with Atg16L1 to form Atg5-Atg12-Atg16L1. Then, this complex further prolongs the phagophore and is required for the LC3 lipidation [39]. When autophagy is triggered, cytosolic LC3 is cleaved to form LC3I. Then, LC3I binds to phosphatidylethanolamine to become LC3II which is recruited to the membrane of the autophagosome. The ratio of LC3II to LC3I is used to assess autophagy levels [40]. Then, the p62 bind to LC3 and deliver specific organelles and protein aggregates to autophagosomes for degradation [41]. Finally, the polyubiquitin-binding protein p62 is degraded by autophagy [42]. Here, we found that the expression of Atg5, beclin1, and LC3II mRNA levels and the AOD of LC3II were increased, but the protein levels of p62 were upregulated in the HS group compared to the CON group. These results indicated that increased autophagosome formation was not caused by autophagy but rather by a decrease in autophagosome clearance, as reflected by accumulation of p62. The significant decrease of the lysosomal transmembrane proteins-related genes of LAMP1 also proved this point. However, ALC treatment effectively promoted the mRNA and protein levels of beclin1 and LC3II, increased the AOD of LC3II and expression of lysosomal biogenesis-related genes (Mcoln1, LAMP1, and LAMP2) in GC-1 cells but decreased the protein levels of p62 under heat stress. These data suggested that the ALC could promote the recovery of GC-1 cells after heat stress by increasing the level of autophagy. Autophagy and apoptosis are regulated by the same regulatory genes, so there is a crosstalk between them [43]. Bcl-2 is one of the regulatory genes. Bcl-2 can bind to Beclin1, prevent the association of Beclin-1 with PI3K complex, and inhibit autophagy [44]. However, the phosphorylated Bcl-2 loses its ability it inhibits autophagy [45]. In this study, we found the Bcl-2 and Beclin-1 were increased, but the levels of autophagy were increased in the HS-ALC group compared to the HS group. So we speculated that phosphorylated Bcl-2 may be involved in the regulation of autophagy in GC-1 cell treatment with ALC after heat stress. More detailed studies of the phosphorylation of Bcl-2 will be needed to obtain a better understanding of the crosstalk between autophagy and apoptosis.

In summary, our research provides the first evidence that ALC can promote GC-1 cells recovery after heat stress by reducing oxidative stress and upregulating autophagy.

## Figures and Tables

**Table 1:** Specific primers used for real-time PCR.

Gene name	Forward primer sequences (5′ →3′)	Reverse primer sequences (5′ →3′)
GAPDH	CCACCAACTGCTTAGCCCCC	ATTCCTGGACCCAAAACGCT
Atg5	ACAAGCAGCTCTGGATGGGACT	CCGCTCCGTCGTGGTCTGATAT
Beclin1	TGATCCAGGAGCTGGAAGAT	CAAGCGACCCAGTCTGAAAT
LC3II	TGTCCACTCCCATCTCCGAAGT	TTGCTGTCCCGAATGTCTCCTG
Bax	TCCACCAAGAAGCTGAGCGAGT	CAGGGCCTTGAGCACCAGTTTG
Bcl-2	ACCGTCGTGACTTCGCAGAGAT	TCTCCCTGTTGACGCTCTCCAC
Caspase3	TGGAGGCTGACTTCCTGTATGC	ATTCCGTTGCCACCTTCCTGTT
Caspase9	TCCTGGTACATCGAGACCTTG	AAGTCCCTTTCGCAGAAACAG
Mcoln1	TGGTGCTGAGCCTCTTCATTGC	ACTGCCACGACGGAACTTGC
Clcn7	AGGCGAGAGAAGGTTGGCATCA	GCTGGCTGGGTGTCTCCTACAT
Lamp1	CGAGTGGCAACTTCAGCAAGGA	CAGCAGGCAGGTTCCGTTGTT
Lamp2	GCAGTACCTGACAAGGCGACAC	CACAGCCCAAGAGACAGCGAAT
Ctsb	GCAGGCTGGACGCAACTTCTAC	TCACCGAACGCAACCCTTCCT
Ctsd	CGGCGTCTTGCTGCTCATTCT	ACACTGGCTCCGTGGTCTTAG

## Data Availability

The data used to support the findings of this study are available from the corresponding author upon request.
